# The case for computational and artificial intelligence-based approaches for sustainable functional food systems for sub-Saharan Africa

**DOI:** 10.3389/fnut.2025.1660245

**Published:** 2025-09-04

**Authors:** Iswat Oyindamola Osundiji, Mayowa A. Osundiji

**Affiliations:** ^1^Department of Pure and Applied Chemistry, Osun State University, Oshogbo, Osun State, Nigeria; ^2^Department of Clinical Genomics, Mayo Clinic, Phoenix, AZ, United States

**Keywords:** functional, food, Africa, computational, sustainable, artificial intelligence

## Introduction

The need for novel approaches that may help drive sustainable food systems, while still retaining high nutritional quality, continues to grow in many parts of the world (1–3), including sub-Saharan Africa (SSA) (4, 5). In particular, the development of sustainable food systems is crucial to help address the challenges of food insecurity in SSA (6–10). SSA has struggled with the negative implications of malnutrition [macro and micronutrient deficiencies] on health (10) and the quality of life of its inhabitants (11), which has impacted socioeconomic development (12). These struggles have been borne by many countries in SSA (6, 13–15), including some nations where populations are expected to continue to grow exponentially over the next few decades (16). Accordingly, the development of healthy and sustainable food systems to address macro and micronutrient deficiencies in SSA has continued to stimulate growing interests (16–19).

Computational and artificial intelligence (AI)-based approaches offer unique opportunities for modeling, developing, and testing food components to help optimize and streamline production processes (20–24) as well as to simulate and assess the efficiency of food distribution (25–27) to enhance sustainability (28, 29), in the context of local climate (30, 31) and socioeconomic challenges (32, 33) in SSA. There are ongoing efforts aimed at harnessing computational biology approaches to mitigate the risk of food insecurity in SSA. These include the African BioGenome Project (AfricaBP) (34, 35), the Southern Africa Network for Biosciences (36). With advances in genomics and genetic engineering, opportunities for computational genomics in SSA are also increasingly becoming topical (37).

Although computational genomics and AI-driven strategies (24, 38) that may enhance the sustainability of staple food systems are increasingly being developed for SSA (39), there is also a critical need for affordable functional food in SSA. Micronutrient deficiency is a huge, but potentially preventable, public health concern in SSA (40–42). Functional foods hold the premise of enhancing health and/or preventing diseases, including illnesses that stem from micronutrient deficiencies (43). There is a growing need for strategies that can help harness the benefits of functional foods as a primary prevention approach to malnutrition to optimize the health of the population across the age continuum in SSA (44). Functional foods are increasingly being made from indigenous plants (such as *Moringa oleifera, Adansonia digitata*, and *Eragrostis tef*) to help address the mounting needs (45–49). Computational modeling [for instance, molecular docking (49, 50)] and AI-powered methods are gradually being utilized to optimize the functional food industry in SSA (49). This opinion article underscores the potential benefits of the upcoming computational and AI-guided approaches in the burgeoning functional food industry in SSA. This report also highlights some of the limitations of computational and AI-guided approaches, considering the socio-economic and infrastructure realities of SSA.

## The growing need for sustainable functional food systems in SSA

Functional foods, also called nutraceuticals, have beneficial health effects in addition to the apparent nutritional benefits (51). With the socioeconomic challenges and health disparities that occur across many countries in SSA, the benefits of functional foods are yet to be fully harnessed (41, 52, 53). Cornucopias of bioactive compounds that are derivable from plant-based products [(PBP), including fruits, vegetables, legumes, and starchy foods] and animal-based products [(ABP, such as collagen, fish oil, and lanolin)] are increasingly being harnessed for preventative and therapeutic purposes in developed countries (54, 55).

Accumulating evidence suggest that functional foods improve gut health, reduce inflammation, and enhance immune function (56, 57). Similarly, functional foods may help prevent chronic diseases such as thrombotic (cerebro and cardio) vascular diseases (58), cancer (50), and type 2 diabetes mellitus (55). The PBP and ABP market sectors are rapidly growing in SSA, providing opportunities to improve the knowledge of consumer perceptions and to identify novel factors that may impact sustainability (59, 60). Given the myriad challenges associated with functional food intake in SSA (41, 53), novel approaches to enhance the functional properties of nutraceuticals, while also limiting production costs, are topical.

## Computational modeling for sustainable functional food systems in SSA

Computational modeling and AI-driven strategies create opportunities for increased use of locally available, amenable, and potentially underutilized crops to expand the sources for functional food products. These include African nightshade (*Solanum scabrum*), Spider plant (*Cleome gynandra*), amaranth (*Amaranthus cruentus*), cowpea (*Vigna unguiculata*), Ethiopian kale (*Brassica carinata*), and common kale (*Brassica oleracea*) (61). For instance, bioinformatic and genomic tools are being applied for the domestication of *Cleome gynandra*, a potential power crop for SSA (36). *C. gynandra* is a leafy vegetable (62) that is a rich source of essential micronutrients, which can be harnessed to produce functional foods to help manage and prevent micronutrient deficiencies (36, 63).

Computational biology and genomic technologies also provide opportunities to mitigate the risks of poor seed quality, crop pests, and diseases, which can result in low yields for power crops, which are crucial for functional food production, as well as ways to reduce food wastage. Recent studies are increasingly suggesting that computational modeling (64, 65) and AI-based approaches may create opportunities for sustainable alternatives to optimize production processes for functional foods. For instance, Feng et al. (65) recently presented an eco-friendly amyloid-like protein coating strategy for perishable fruit preservation using computer-aided molecular simulation that may pave the way for sustainable approaches for mitigating wastage of functional food in SSA. Additionally, Hu et al. applied computational tools to propose sustainable alternatives for the synthesis of chiral cyclohexenones, which are critical components of many functional food products (64). Similarly, Asfha et al. applied a computational modeling approach, known as molecular docking, to show the potential benefits of a staple crop in SSA, *Eragrostis teff* [commonly known as teff], in the prevention of osteoporosis (49).

## Discussion

There is a growing need for affordable functional food in SSA to help improve the health of the population (41, 53). Considering that agricultural productivity in SSA is constrained by climate variability (66) and limited infrastructure (67), computational modeling and AI-powered approaches offer targeted solutions for optimizing indigenous food systems to address nutritional deficiencies. Computational modeling (64, 65) and AI-based approaches (29, 68) may create opportunities for sustainable alternatives to optimize production, as well as storage and distribution processes for functional foods.

The potential for computational modeling and AI-enabled strategies to facilitate functional food ingredient discovery and characterization (68) as well as safety (29) continues to hold great potential for enhancing the growth of nutraceuticals in SSA's diverse ecosystems. Nevertheless, the adoption and utilization of computational and AI-assisted modeling for sustainable food systems are not without challenges and limitations (69, 70). These include the potential costs of computing infrastructure, availability of adequate internet access with high-quality data streaming [which is crucial for real-time analytics and machine learning], access to technical expertise, funding for training costs and computing infrastructure, data integrity and validation, as well as support from multi-sectoral stakeholders (including government, private sector, academia, and non-governmental organizations) (38).

To leverage the benefits of computational modeling and AI-powered strategies for the growth of functional food systems in SSA, multi-stakeholder partnerships (involving government, industrial, and academic representatives) are increasingly needed. Funding and policy incentives that can support computational modeling and AI-powered strategies for functional food systems in SSA will be beneficial. It is hoped that the increasing applications of computational modeling and AI-driven technologies will help foster the development of sustainable functional food systems across SSA.
